# A rare coexistence: Poland’s syndrome and cardiac angiosarcoma

**DOI:** 10.1186/s13019-023-02138-z

**Published:** 2023-01-18

**Authors:** Fatih Kizilyel, Rafet Gunay, Mehmet Rum, Mehmet Yilmaz, Bulend Ketenci

**Affiliations:** Department of Cardiovascular Surgery, Dr SiyamiErsek Thoracic and Cardiovascular Surgery Training and Research Hospital, Tibbiye Cd No 13, Haydarpasa, 34668 Uskudar, Istanbul, Turkey

**Keywords:** Cardiac angiosarcoma, Poland's syndrome, Neo-atrium, Cardiac oncology

## Abstract

Poland’s syndrome, a rare genetic disorder that accompanies malignancies, musculoskeletal disorders, cardiac and genitourinary syndromes. There is no study that represents the association between cardiac angiosarcoma and Poland’s syndrome. A 24-year-old female patient previously diagnosed with Poland’s syndrome was admitted to our hospital complaining of dyspnea. Diagnostic imaging showed an irregular mass in the right atrial cavity. After successful surgery, she was discharged uneventfully and the 3rd month oncologic follow-up reveals none of residual mass. The coexistence has not been diagnosed and treated in a cardiac surgery department before. With this presentation, we aimed to contribute to the literature with this presentation, for the right and early diagnosis and management of possible new cases in the future can be diagnosed and treated correctly and early.

## Introduction

Poland's syndrome is a genetic disorder of unknown origin characterized by the absence of the pectoralis major muscle. The incidence of Poland’s syndrome ranges from 1 in 7,000 to 1 in 100,000 live births. Congenital anomalies including upper limb malformations have been reported; cardiac and urologic disorders may also be associated with the syndrome [1]. Cases of Poland’s syndrome associated with leukemia and carcinoma confirm the association between developmental defects and tumors and require oncologic awareness.

Primary cardiac neoplasms are rare malignant tumors. Benign myxomas constitute the majority of them. Of the remaining 25% of tumors, angiosarcomas are one of the subtypes. [2] In this study, we would like to present a case of rapidly progressing cardiac angiosarcoma, surgical management and postoperative follow-up in a 24-year-old female patient with Poland’s syndrome.

## Case report

**Fig. 1:**
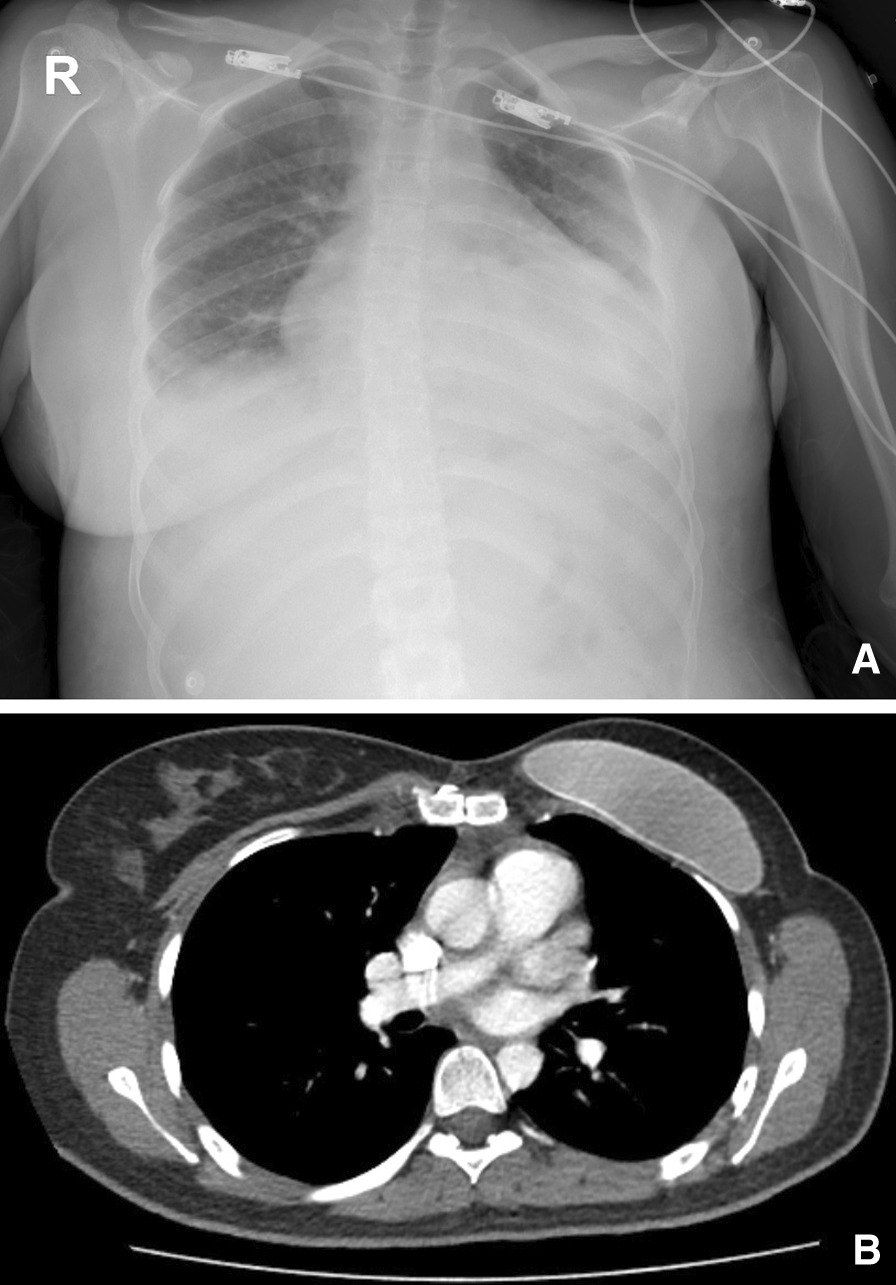
**A** Preoperative chest X-Ray showing absence of left breast tissue. **B** Postoperative CT- angiography showing absence of left pectoralis muscle and left artificial breast

**Fig. 2:**
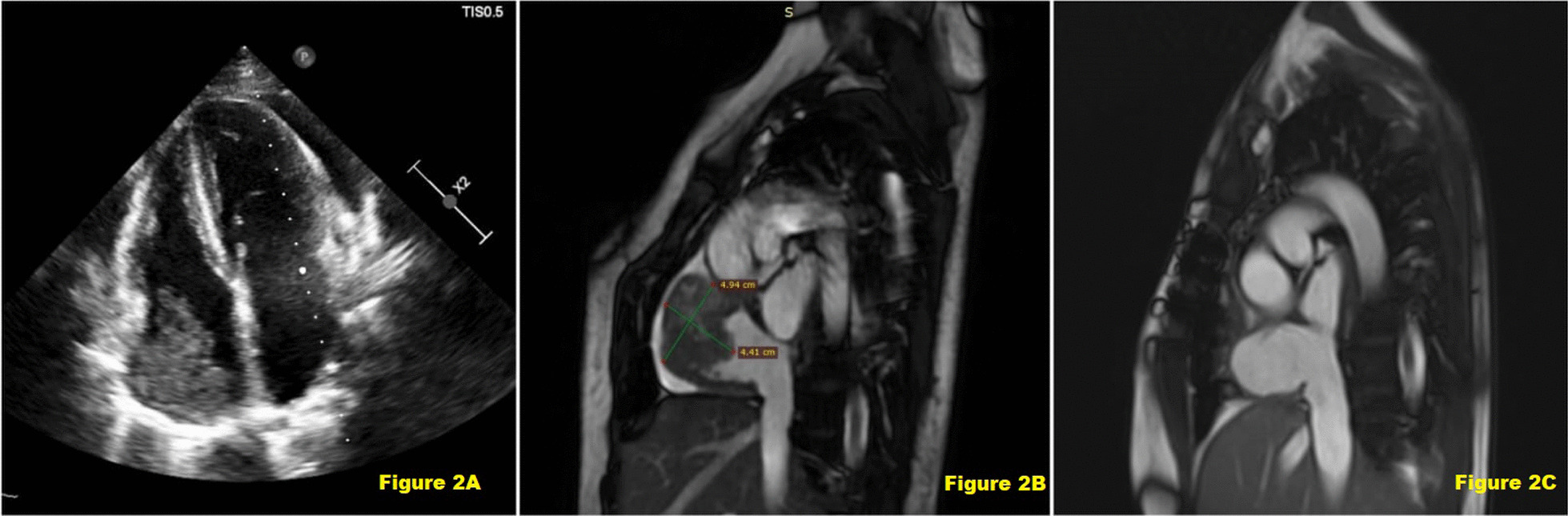
**A** Preoperative echocardiographic image shows a mass in the right atrium. **B** Cardiac MRI image that showing an angiosarcoma with a diameter of 4.9 × 4.1 cm (preoperative). **C** Cardiac MRI image showing Neo-Atrium postoperative 3rd month

A 24-year-old female patient was admitted to our clinic with complaints of dyspnea and palpitations. Her medical history included Poland's syndrome and she had an artificial left breast prosthesis inserted 3 years ago. (Fig. 1A and B) Physical examination was unremarkable except for dyspnea; vital signs were stable. Chest X-ray showed pleural effusion and cardiomegaly, and transthoracic echocardiography (TTE) revealed an ejection fraction of 60%, a massive lesion in the right atrium, pericardial effusion causing cardiac tamponade of 2.3 cm lateral to the right ventricle and 3 cm lateral to the left ventricle, without tricuspid regurgitation (Fig. 2A).

The patient was admitted to the intensive care unit and pericardiocentesis was performed. 730 ml hemorrhagic fluid was drained and then bilateral pleurocan was inserted because of pleural effusion. 1300 cc drainage was achieved. Contrast-enhanced computed tomography angiography (CTA) demonstrated a mass lesion size of 39 × 42 mm, extending towards the superior vena cava in the right atrium appendix and the superior part of it. On the 3rd day of her admission, (18)F-FDG positron emission tomography reveals that the right atrium mass was 44*48 mm in size, contained central necrosis, invaded the mediastinal fatty planes, and no systemic metastases were detected (SUVmax: 17.9). Cardiac magnetic resonance imaging (MRI) was performed to better visualize tumor anatomy and morphology (Fig. 2B). Because of the aggressive enlargement of the tumor, immediate surgery was planned.

**Fig. 3:**
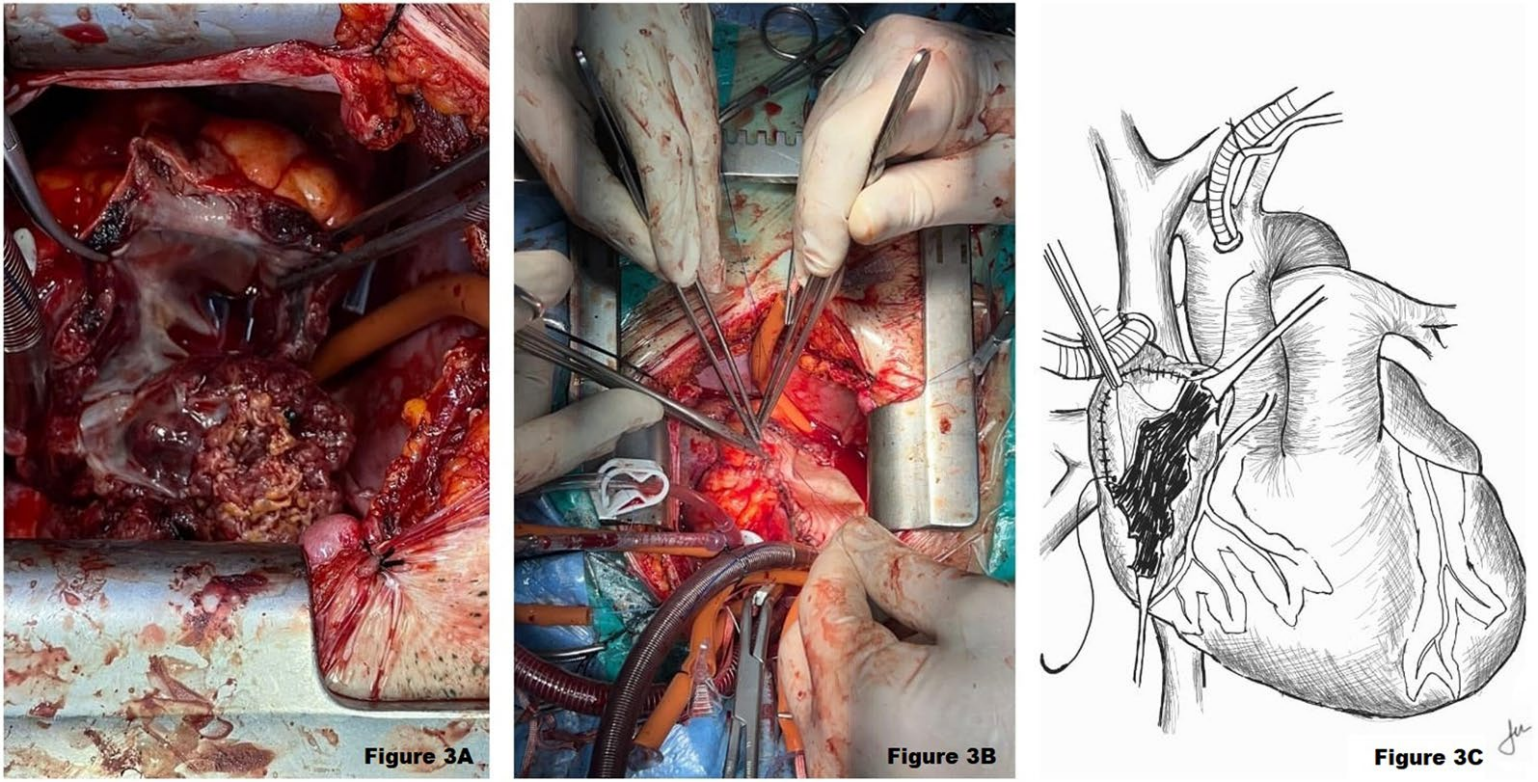
**A** Image of operative field after right atriotomy, angiosarcoma is seen, **B** After resection of the mass, the right atrial cavity is enclosed with a bovine pericardial patch to form Neo-Atrium, **C** Schematic representation of the surgical procedure, anastomosis of the pericardial patch with the free edge of the right atrium

After median sternotomy, arterial cannulation of the ascending aorta and venous cannulation of the superior vena cava—right femoral vein, followed by cardiopulmonary bypass. After right atriotomy, a 5-cm-diameter mass was resected with its associated free atrial wall (Fig. 3A). A residual mass on the inferior vena cava ostium was also resected. Involved segments of the right ventricle were partially resected. The superior vena cava and tricuspid valve were not involved. After complete resection of the native right atrium, Neo-Atrium formation with a bovine pericardial patch was performed (Fig. 3B). The operation was successfully completed.

**Fig. 4:**
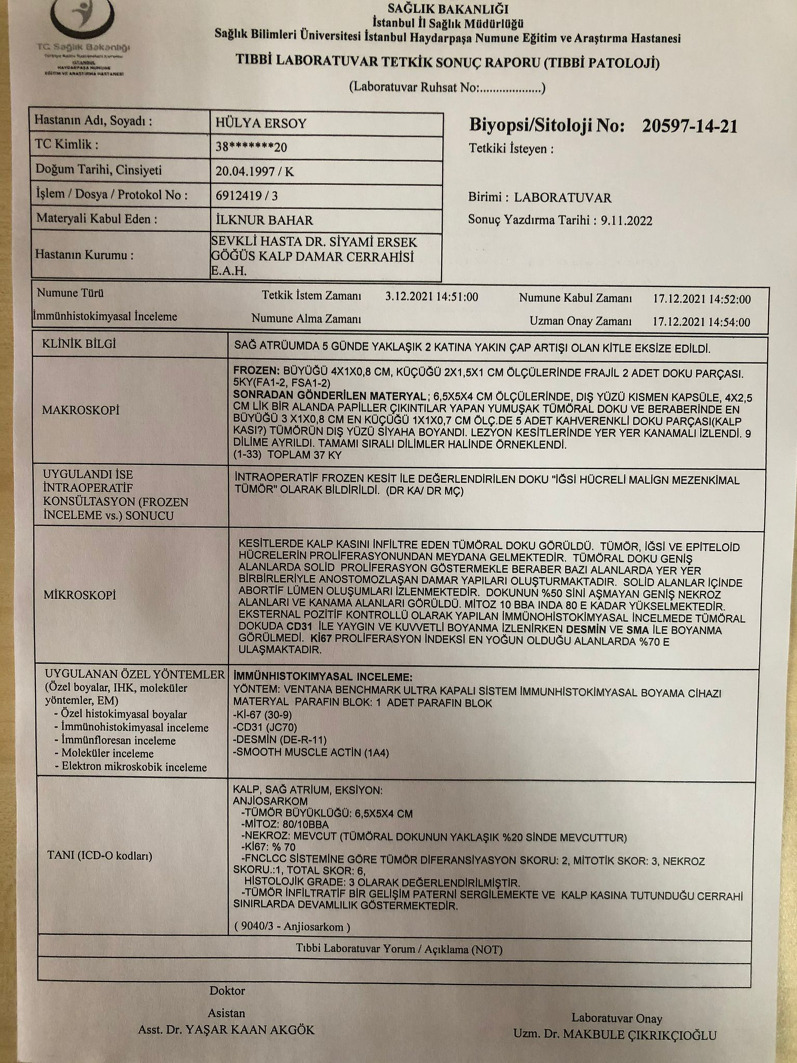
The report that was requested from the pathology department shows evaluation of the specimens

Postoperative transthoracic echocardiography showed an ejection fraction of 55% and mild pericardial effusion. She was discharged after a successful, uneventful follow-up. The patient's cardiac symptoms resolved. After oncologic evaluation according to the pathologic specimens taken at surgery (Fig. 4), the diagnosis of angiosarcoma was corrected and the remaining adjuvant chemotherapy was scheduled for three sessions. (Doxorubicin, Mesna) After the 3rd dose, a control MRI angiography was performed, which revealed no residual mass (Fig. 2C). Follow-up continued without active symptoms.

## Discussion

Poland's syndrome is an inherited disorder that results from local mesoderm defects in the embryonic period. Although the cause of Poland syndrome remains unknown, there is evidence that a genetic factor may be involved. However, no genes responsible for the disease have yet been identified. The syndrome may be associated with malformations of the musculoskeletal system, abnormalities of the genitourinary system, and cardiac abnormalities. Twelve cases of leukemia and three cases of lymphoma have been reported [3], and coexistence with solid tumors has also been reported [4]. This association between developmental abnormalities and tumors represents oncologic awareness in patients with Poland’s syndrome.

Primary cardiac tumors are extremely rare; the incidence documented in series is 0.0001–0.03% [5]. Most of these tumors are benign (75%), and of the malignant tumors, primary cardiac angiosarcoma is the most common histologic subtype [6].

Angiosarcoma is a very aggressive neoplasm that originates from the endothelium and has a high risk of local recurrence and systemic metastasis [7]. Half of the cases occur in the head and neck region. Parameters such as primary site, metastases, and grade may indicate a poor prognosis. Surgery, chemotherapy, and radiation are the main treatment modalities. The male to female ratio for primary cardiac angiosarcomas has been documented as 2–3/1.

Cardiac angiosarcomas usually originate from the right atrium and adjacent structures and may cause congestive heart failure, pericardial effusion, and cardiac tamponade, depending on location and degree of invasion [8]. Symptomatology and prognosis also depend on localization and invasion. It can occur in the cardiac cavities, as in the present case, as well as in the main vessels and cardiac valves, affecting the normal physiology of the heart [9]. The rarity of this diagnosis has led to a search for possible new associations in clinical practice and has complicated the standardization of therapy. It is a malignant disease with a poor prognosis and patients usually die months after diagnosis.

Patients often present with nonspecific constitutional symptoms such as weight loss, shortness of breath, and anemia. Depending on the location and invasion, chest pain, palpitations, dyspnea, etc. may also be present [10].

The most important technique in diagnosis is echocardiography. In addition to transthoracic echocardiography, especially in posteriorly located masses, transesophageal echocardiography also provides information about location, size, adhesion, and association with adjacent structures [11]. Computed tomography provides more detailed information about the characteristics of the mass and systemic involvement, and MRI is used with regard to the morphologic appearance and invasion of the mass. Positron emission tomography (PET-CT) is routinely used for the diagnosis and treatment of metastases and for follow-up [12].

Metastases are usually present at the time of diagnosis. The most common site is the lung, but liver, lymph node, bone, and brain metastases may also occur. [13]

Surgery is the most commonly chosen treatment modality, especially for localized disease. In metastatic and widespread cases, partial resection may be helpful in relieving symptoms. Patients have been reported to die 9–12 months after a diagnosis without surgical treatment [14]. Wide resection is critical to the success of surgery and, when successful, has a fairly positive effect on survival, depending on the relationship of the tumor to adjacent tissues.

Although cardiac angiosarcomas are usually resistant to chemotherapy and radiotherapy, adjuvant chemotherapy may help reduce metastatic tumor size [15]. Cisplatin, cyclophosphamide, dacarbazine, doxorubicin, ifosfamide, and paclitaxel are agents commonly used for treatment [16].

Cardiac angiosarcomas are malignant tumors with a poor prognosis that are difficult to treat in various locations, close to vital structures, and with a high rate of metastasis. Survival can be achieved by patients who are diagnosed early, especially in the localized form of the disease.

## Conclusion

Cardiac angiosarcomas are rare malignant tumors, depending on their location, and mortality is extremely high because of their aggressive behavior. Surgical resection seems to offer the best prognosis, besides adjuvant chemotherapy and immunotherapy may help in the limited population.

Previous studies and reviews have focused on cardiac angiosarcoma as a whole. The aim of this paper was to consolidate information on possible associations with this rare genetic disorder to increase the likelihood of early diagnosis.

This is the first report of a case in which Poland’s syndrome coexists with cardiac angiosarcoma. As in our case, cardiac angiosarcoma is a rapidly progressive pathology and prompt intervention should be made. As is known in the literature about Poland syndrome, patients should be included in the screening program for possible concomitant malignancies.

## Data Availability

All the data and materials can be found from our clinic’s local database “NUCLEUS” as it recommended.
